# Effects of Early Stress Exposure on Anxiety-like Behavior and *MORC1* Expression in Rats

**DOI:** 10.3390/biom14121587

**Published:** 2024-12-12

**Authors:** Annakarina Mundorf, Nadja Freund

**Affiliations:** 1 Division of Experimental and Molecular Psychiatry, Department of Psychiatry, Psychotherapy and Preventive Medicine, LWL University Hospital, Ruhr University Bochum, 44801 Bochum, Germany; 2 Institute for Systems Medicine and Department of Human Medicine, MSH Medical School, 20457 Hamburg, Germany

**Keywords:** maternal separation, social isolation, depression, development, rt-PCR, prefrontal cortex

## Abstract

Exposure to stress during early and late childhood can lead to long-lasting neurobiological and behavioral impairments. Although sensitive periods for stress exposure are well established, less is known about the trajectory of induced alterations throughout development. In this study, we investigated the impact of maternal separation (MS), social isolation, and their combination on anxiety-like behavior and gene expression across developmental stages. Sprague Dawley rats were exposed to one or both stressors and later assessed for anxiety-like behavior in juvenility, adolescence, and adulthood. mRNA levels of *Morc1*, a gene linked to early-life stress and depression, were measured in the medial prefrontal cortex to assess developmental changes. The results showed that MS had age- and sex-dependent effects on anxiety-like behavior. Juveniles exhibited less anxiety after MS, while adolescents showed more pronounced behavioral changes following social isolation. No behavioral changes were observed in adults. Males exhibited greater anxiety-like behavior than females in adolescence and adulthood, but not in juvenility. Female adults exposed to both MS and social isolation had significantly lower *Morc1* expression compared to controls. These findings highlight the dynamic effects of early stress across the lifespan, underscoring the critical role of adolescence and differential stress susceptibility by age and sex.

## 1. Introduction

Generally, stress exposure elicits adaptive psychological and physiological responses. Psychologically, stress can affect the individual’s mental state, alter mood, arousal, and motivation, and even influence subjective well-being. The body responds to stress exposure by increasing glucocorticoid levels that affect the hypothalamic–pituitary–adrenal (HPA) axis, one central stress response system determining the effects of stress exposure on the brain [1,2,3,4]. During sensitive brain development and maturation phases, severe stress exposure can induce neuroanatomical changes, which may result in, e.g., reduced hippocampus, corpus callosum, amygdala, or prefrontal cortex volumes [5]. Brain regions especially vulnerable to permanent stress-induced alterations show a prolonged maturation until childhood and adolescence [4]. Consequently, traumatic stress in early life can lead to long-lasting neuronal changes that are linked to impairments in cognitive functions like memory and emotional perception [5,6,7].

Eventually, the most critical period in terms of stress exposure is early childhood, as early exposure alters susceptibility to the pathogenic effects of stress throughout life [8]. However, experiencing multiple hits of adversity has profound effects as well. This has been reflected in the diathesis–stress model of depression, also known as the two-hit model, which suggests that early-life adversity (first hit) makes the brain vulnerable. In contrast, subsequent stress (second hit) triggers psychopathology [9,10]. This assumption is consistent with the consequences that cumulative stress leads to more severe symptoms [11,12]. It is also hypothesized that each new stress exposure or psychiatric episode activates neurotoxic pathways, resulting in brain structure decline as the disorder progresses [13]. Furthermore, longitudinal studies suggest that individuals who experience adversity early in life are more likely to be repeatedly exposed to adverse events [14]. Thus, it is interesting to investigate the consequences of stress exposure during different periods and the consequences of cumulative stress.

In addition, specific genetic risk variants and epigenetic modifications are found to interact with early-life stress, resulting in psychopathology [15,16,17], indicating that a gene x environment interaction influences the etiology of psychiatric disorders. One promising candidate gene to mediate between early exposure and psychopathology, which has been associated with both early-life stress and depression in rodent and human studies, is the *MORC family CW-type zinc finger 1* (*MORC1*) gene [18,19,20,21]. Nieratschker and colleagues were the first to report an association between reduced *MORC1* methylation and early-life stress in human umbilical cord blood, primate blood, and rat brain tissue. Furthermore, they found a link between single-nucleotide polymorphisms in the *MORC1* gene and major depression using a genome-wide association study database [20]. To further disentangle the role of the *MORC1* gene in the development of depression, *MORC1* methylation patterns of buccal cells were examined in healthy participants [19]. To capture depressive symptoms, the Beck Depression Inventory was completed by the participants. As a first result, increased depressive symptoms were associated with increased *MORC1* methylation [19]. Increased *MORC1* methylation was associated with neurite orientation dispersion and density markers and a volume reduction in the hippocampus and medial prefrontal cortex [18]. To also investigate whether early stress, in this case, is defined as complications during birth, the participants were also asked to report any complications such as preterm birth, prolonged labor, and multiple or caesarian births. In this case, participants reporting higher numbers of complications showed a decreased *MORC1* methylation, similar to the results found by Nieratschker et al. [19,20]. A multicentric study, eager to identify the role of altered *MORC1* methylation patterns in depression and early adversity, analyzed whole-blood DNA methylation in depressed patients and matched healthy controls. In this study, a history of childhood trauma was defined as early adversity and recorded. In all three cohorts included, increased *MORC1* methylation correlated with increased depressive symptoms. Having a history of childhood trauma was not associated with altered *MORC1* methylation [21].

DNA methylation is associated with gene expression [22,23]. Therefore, to reveal the functional involvement of *MORC1* methylation in the development of psychopathologies, Nieratschker and colleagues investigated *MORC1* mRNA gene expression in cord blood. However, they did not see an effect of early-life stress on gene expression matching the observed methylation changes [20]. *Morc1* gene expression has recently been studied in the rodent brain [24]. The study revealed that *Morc1* mRNA is expressed relatively stable in rats throughout different stages of development and that *Morc1* mRNA is found in several brain regions of mood regulation such as the medial prefrontal cortex (mPFC), amygdala, hypothalamus, nucleus accumbens, and hippocampus [24]. Mice lacking the *Morc1* gene display depressive-like behavior [25]. Additionally, reduced mPFC *Morc1* mRNA levels were evident in dams after stress exposure in the vulnerable postpartum time [26].

These findings raise the question of whether the reported altered methylation of the *MORC1* gene after early-life stress is reflected in altered mRNA expression in the brain. Furthermore, it is unclear in which ways the observed hypomethylation after early-life stress and the hypermethylation associated with depression both result from early adversity or whether these modifications occur independently. To this end, the contrariwise modifications need to be disentangled, and changes in gene expression level should be investigated over the course of development.

An established model to induce early-life stress in animals is the maternal separation (MS) paradigm. In this paradigm, pups and dams are separated (sometimes the pup is also separated from its littermates) over several hours/day in the early postnatal period [27,28]. Different neurobiological and behavioral alterations develop in the pups, depending on the postnatal days and hours chosen for separation [29,30]. MS in rats induces, e.g., depressive-like behavior [31,32], anxiety-like behavior [33], and increased reward-seeking behavior [34]. Moreover, MS leads to decreased neuronal plasticity [35,36]. However, the MS paradigm is a pre-weaning stressor, as separation from the dam is most effective until the pups are weaned. Thus, MS induces stress in the pups’ early childhood. Social isolation (ISO) can be seen as a similar post-weaning stressor, allowing one to investigate the consequences of stress exposure during late childhood. In this paradigm, the experimental animal is singly housed over a distinct period (days or weeks), whereas the controls stay in group housing. Thereby, ISO leads to similar neurobiological alterations and induces anxiety-like behavior and depression-like phenotypes as MS [37,38]. Both stressors induce stress mainly via socially isolating the animal. Therefore, they are often used alternately or consecutively, enabling experimenters to investigate the effects of sensitive periods or cumulative exposure [39,40,41,42,43].

Early-life stress induces changes on the behavioral level as well. When focusing on symptoms developing throughout development, altered anxiety-like behavior is the preferred phenotype, especially as anxiety disorders are one of the first to develop in childhood [44]. In rodents, anxiety-like behavior is commonly assessed using the elevated plus-maze and the marble burying test [45,46,47,48,49].

As mentioned earlier, the PFC is a region of interest in early adversity. This region develops over a long period and is vulnerable to stress-induced disruptions during childhood and adolescence [4,50]. In particular, the medial part of the prefrontal cortex (mPFC) is a key structure associated with the pathophysiological processes contributing to depression and anxiety disorders (for review see [51]). Considering the time of exposure planned in this study, the stress-induced changes in gene expression should be most distinct in the mPFC.

This study aimed to examine the consequences of early stress exposure on gene expression and behavior during critical windows of brain development.

## 2. Materials and Methods

### 2.1. Animals

Thirty-two pregnant Sprague Dawley rats (Charles River Laboratories, Sulzfeld, Germany) were singly housed in standard Makrolon IV cages upon arrival between gestational days 13–15. The animals were housed under standard conditions (22 ± 2 °C room temperature, 55 ± 25% humidity) and standard lighting (12 h/12 h) with free access to water and food. Offspring were either group-housed with same-sex littermates or single-housed. The day of birth was considered postnatal day 0. On postnatal day 2, all pups were sexed and culled to 10 per litter when necessary (if possible, five females and five males). The rats were weaned at postnatal day 21. All experiments were conducted with relevant guidelines and regulations under the principles of Germany’s Animal Welfare Act after approval by the LANUV (Landesamt für Natur, Umwelt und Verbraucherschutz North Rhine-Westphalia) on 17 October 2016 and 29 May 2018. The pregnant rats were randomly assigned to MS or controls (CRL) by an independent party. The animals were tested in two cohorts. In cohort 1 (8 MS dams and 8 CRL dams), the offspring were behaviorally tested and sacrificed in juvenility and adolescence, respectively. In cohort 2 (8 MS dams and 8 CRL dams), the offspring were behaviorally tested and sacrificed in adulthood. To avoid litter effects, one animal per sex and group was taken per litter. However, as one animal (female CRL) could not be included due to technical problems during behavioral testing, another female CRL from one of the other litters was tested and included. Four pregnant dams (2 MS and 2 CRL) and later their offspring were treated and/or tested simultaneously. The sample size was determined using G*Power statistical software 3.1 [52]. One day after behavioral testing, the animals were deeply anesthetized with an intraperitoneal injection of a ketamine (100 mg/mL; cp-pharma, Burgdorf, Germany) and xylazine (20 mg/mL; cp-pharma, Burgdorf, Germany) mixture (ratio of 2:1) and sacrificed by decapitation. The juveniles were sacrificed at postnatal day 22, the adolescents at postnatal day 42, and the adults at postnatal day 62 in the morning. Brains were extracted and stored at −80 °C until further use. For dissection, the whole brain was placed on an upside-down Petri dish filled with dry ice. Then, the mPFC was dissected at bregma 2.20 mm according to Paxinos and Watson’s *The Rat Brain in Stereotaxic Coordinates* [53] and as previously described [24,26].

### 2.2. Stress Exposure

**Figure 1:**
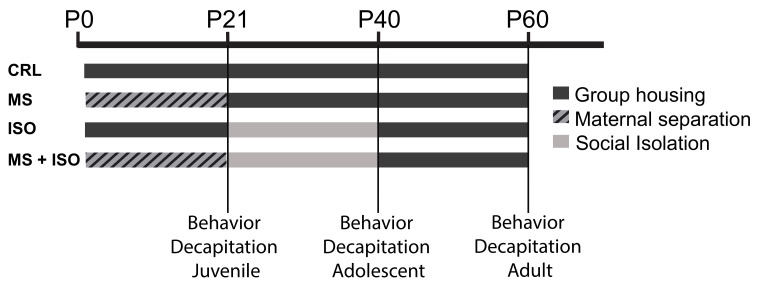
Schematic timeline of the experimental design and stress exposure. P: postnatal day; CRL: controls; MS: maternal separation; ISO: social isolation. At P21, P40, or P60, anxiety-like behavior was tested (over 2 days for adolescents + adults), followed by decapitation on the next day.

MS was conducted as previously described [54,55]. In brief, pups were separated from the dam and littermates for 4 h daily over postnatal days 2–20, during the dark (=active) phase. The start of separation was kept constant at around 11:30–12 am. For separation, the pups were placed in separate cages. Until postnatal day 10, when the pups could heat themselves, they were kept on a heating mat adjusted to 37 °C with home cage bedding, separated from littermates by a self-made plastic grid that allowed auditory and olfactory but no physical contact. CRL pups were only separated from dams to be weighed every 4–5 days. On the day of weaning at postnatal day 21, one pup per sex and litter was tested for anxiety-like behavior. Again, one animal per litter and sex was single-housed until postnatal day 39, experiencing social isolation (ISO) as a late stressor. The other pups were group-housed with same-sex littermates (in groups of two for males and groups of four for females) until they reached adolescence (postnatal day 39). Animals from the ISO group were group-housed with same-sex ISO animals from postnatal days 40–60. Thus, four groups derive (I) early stress exposure (MS), (II) late stress exposure (ISO), (III) early and late stress exposure (MS + ISO), and (IV) no stress exposure (CRL). Moreover, animals were behaviorally tested as juveniles, adolescents, or adults (Figure 1). For each group, eight animals per sex and stress condition were analyzed.

### 2.3. Anxiety-like Behavior

Anxiety-like behavior was assessed using the elevated plus-maze and the marble burying test. In the elevated plus-maze, the animals were exposed to a maze with four arms (50 cm × 10 cm) 50 cm from the ground. Two arms were closed, providing protection, and two opposing arms were open. The animals were placed in the maze, facing the same closed arm, and observed for 5 min. The time spent in the open and closed arms, respectively, and the total number of open-arm entries (from closed to open and from the left to the right open arm) were manually scored. Time spent in the open was counted when at least both the front paws were on the open arm. When the whole body was in the middle or in the closed arm, this was counted as time in the closed arm. In the marble burying test, 20 marbles were placed symmetrically (4 × 5 rows) in a new standard Makrolon IV cage on smoothened bedding. After a 15 min trial, the number of marbles fully covered with bedding was counted. Juveniles were tested in the elevated plus-maze, followed by marble burying on the same day. Adolescents and adults were tested in the elevated plus-maze, followed by marble burying the next day. Testing was always performed during the dark (=active) phase. All tests were rated by the same experimenter blinded to the condition.

### 2.4. Real-Time PCR mRNA Analysis

Real-time PCR mRNA analysis was performed as previously described [24,56]. In brief, RNA was extracted using the NucleoSpin^®^ TriPrep (Macherey-Nagel, Düren, Germany) with slight modifications. Briefly, 40 μL of RNase-free water was added to each sample to obtain RNA. Then, the concentration and quality of RNA were measured using the NanoDrop™ ND-1000 Spectrophotometer (PEQLAB Biotechnologie, Erlangen, Germany). RNA was converted to cDNA using the High-Capacity RNA-to-cDNA™ Kit (Thermofisher Scientific, Darmstadt, Germany). For real-time PCR analysis, the TaqMan™ Gene Expression Master Mix (Thermofisher Scientific, Darmstadt, Germany) and primers were used. Three different TaqMan gene expression assays were used. *Morc1* (Rn01474745_m1) as the target gene and *Glyceraldehyde−3-phosphate dehydrogenase, Gapdh* (Rn01775763_g1) and *Actin, beta* (Rn00667869_m1) as two housekeeping genes with stable expression in stress conditions [57,58], respectively. A testis sample was used as a reference sample. All samples and genes were assayed in duplicates. Real-time PCR (Applied Biosystems 7500 Fast Real-Time PCR System, Watham, MA, USA) reaction was performed according to the manufacturer’s protocol. Delta CT was calculated as ΔCT = CT *Morc1* − (CT *Gapdh* + CT beta Actin). Then, delta delta CT (2^−^^∆∆CT^) was calculated as 2^−∆∆CT^ = ΔCt (*Morc1*) − ΔCt (housekeeping genes) (as described by [59]). The resulting 2^−∆∆CT^ values were used for statistical analysis. Due to partially low mRNA concentrations or the repeated failure of samples in real-time PCR analysis, several samples had to be excluded for further analyses. The final sample composition is shown in Table 1.

### 2.5. Statistical Analysis

For statistical analysis, GraphPad Prism 10.2.3 (Dotmatics, Boston, MA, USA) was used. For each age group, statistical analysis was conducted separately as the juvenile/adolescent and the adult animals were investigated in separate cohorts. For juveniles, a two-way ANOVA with independent factors sex and MS was conducted for all measurements. Tukey’s multiple comparisons test was used for post hoc analysis. The data of adolescent and adult animals were analyzed by three-way ANOVA with the independent factors sex, MS, and ISO. For post hoc analysis, each sex was analyzed separately using Tukey’s multiple comparisons test. *p*-values below 0.05 were considered significant.

## 3. Results

### 3.1. Juveniles

**Figure 2:**
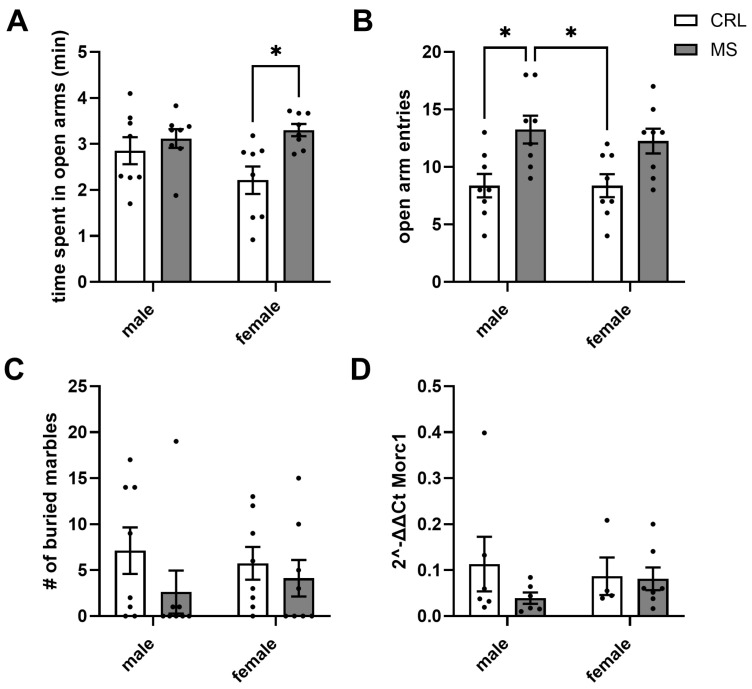
Results for juveniles. The time spent in the open arms of the elevated plus-maze of a total of 5 min with the time given in minutes (**A**) and the number of open arm entires (**B**). The mean number of buried marbles (**C**). Also, the mean mPFC *Morc1* 2^−∆∆CT^ values are given for both groups (**D**). * *p* = 0.01.

The two-way ANOVA revealed a significant effect of MS for the time spent in the open arms (F (1, 28) = 7.8, *p* = 0.009). There were no main effects of sex (F (1, 28) = 0.9, *p* = 0.35) nor an interaction of MS and sex (F (1, 28) = 2.9, *p* = 0.1). Females in the MS group spent nearly 50% more time in the open arms than controls (*p* = 0.018) (Figure 2A). This finding is in line with an effect of MS on the total number of open-arm entries (F (1, 28) = 16.44, *p* = 0.0004), but again, there was no main effect of sex (F (1, 28) = 0.2, *p* = 0.65) or an interaction (F (1, 28) = 0.2, *p* = 0.65). Here, males in the MS group had nearly 60% more open-arm entries than controls (*p* = 0.017) (Figure 2B). The number of buried marbles was not influenced by MS (F (1, 28) = 1.96; *p* = 0.17) or sex (F (1, 28) = 0.0008; *p* = 0.98) (Interaction: F (1, 28) = 0.43; *p* = 0.52) (Figure 2C). Similarly, *Morc1* expression did not differ between MS (F (1, 19) = 1.1; *p* = 0.31) or sex (F (1, 19) = 0.04, *p* = 0.84) (Interaction: F (1, 19) = 0.8; *p* = 0.39) (Figure 2D).

### 3.2. Adolescents

**Figure 3:**
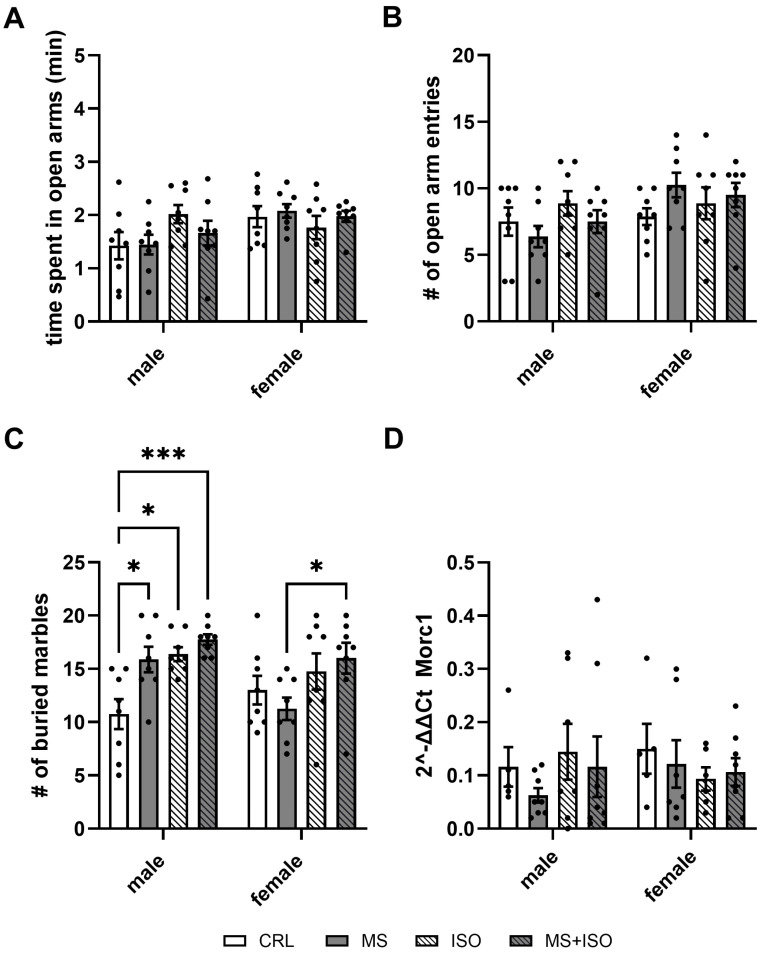
Behavioral and gene expression results for adolescent rats. Time spent in the open arms of the elevated plus-maze of a total of 5 min is given (**A**), as is the total number of open-arm entries (**B**). The mean number of buried marbles after 15 min (**C**) and the mean mPFC Morc1 2^−∆∆CT^ values are given per group (**D**). Mean values and SD are given per group. CRL = controls; MS = maternal separation; ISO = social isolation; MS + ISO = both stressors. * *p* < 0.05; *** *p* < 0.001.

The three-way ANOVA revealed that in the elevated plus-maze, the main effect of sex (F (1, 56) = 5.2; *p* = 0.03) and an interaction of sex and ISO were present (F (1, 56) = 4.2; *p* = 0.046). No main effects of ISO (F (1, 56) = 0.85; *p* = 0.36) and MS (F (1, 56) < 0.001; *p* = 0.98) or other interactions (sex × MS: (F (1, 56) = 1.5; *p* = 0.23); ISO × MS: (F (1, 56) = 0.25; *p* = 0.62); sex × ISO × MS: (F (1, 56) = 0.76; *p* = 0.39)) were found. Overall, females spent slightly more time (18%) in the open arm than males. While ISO increased the time spent in the open arms in males, this was reduced in females after ISO. However, post hoc analysis showed no significant differences between groups (Figure 3A). The number of open-arm entries shows the main effect of sex (F (1, 56) = 5.7; *p* = 0.02) and an interaction of sex and MS (F (1, 56) = 4.4; *p* = 0.04). There were no main effects of ISO (F (1, 56) = 1.1; *p* = 0.3) and MS (F (1, 56) = 0.04; *p* = 0.85) or other interactions (sex × ISO: (F (1, 56) = 0.7; *p* = 0.39); ISO × MS: (F (1,56) = 0.58; *p* = 0.45); sex × ISO × MS: (F (1, 56) = 0.33; *p* = 0.57)). In line with the increased time spent in the open arms, females had about 20% more open-arm entries. While MS did not significantly increase the number of open-arm entries in females, it did not significantly decrease in males following MS (Figure 3B).

The number of buried marbles depended on ISO (F (1, 56) = 16.41; *p* = 0.0002) and an interaction of sex × MS (F (1, 56) = 4.1; *p* = 0.048). No other main effects or interactions were found (sex: F (1, 56) = 2.77; *p* = 0.1); MS: F (1, 56) = 3; *p* = 0.09); sex × ISO F (1, 56) = 0.08; *p* = 0.77); ISO × MS: F (1, 56) = 0.05; *p* = 0.82); sex × ISO × MS: F (1, 56) = 3.8; *p* = 0.056). Post hoc analysis revealed that in males, the controls buried significantly fewer (about 50% fewer) marbles compared to MS (*p* = 0.02), ISO (*p* = 0.01), and MS + ISO (*p* <0.001). In females, the MS group buried 40% fewer marbles than the MS + ISO group (*p* = 0.039) (Figure 3C).

No significant main factors or interactions for the expression of *Morc1* were found in adolescents (sex: F (1, 46) = 0.07; *p* = 0.79; ISO: F (1, 46) = 0.008; *p* = 0.93; MS: F (1, 46) = 0.69; *p* = 0.41; sex × ISO: F (1, 46) = 1.72; *p* = 0.2; sex × MS: F (1, 46) = 0.32; *p* = 0.58; ISO × MS: F (1, 46) = 0.33; *p* = 0.57; sex × ISO × MS: F (1, 46) = 0.33; *p* = 0.57) (Figure 3D).

### 3.3. Adults

**Figure 4:**
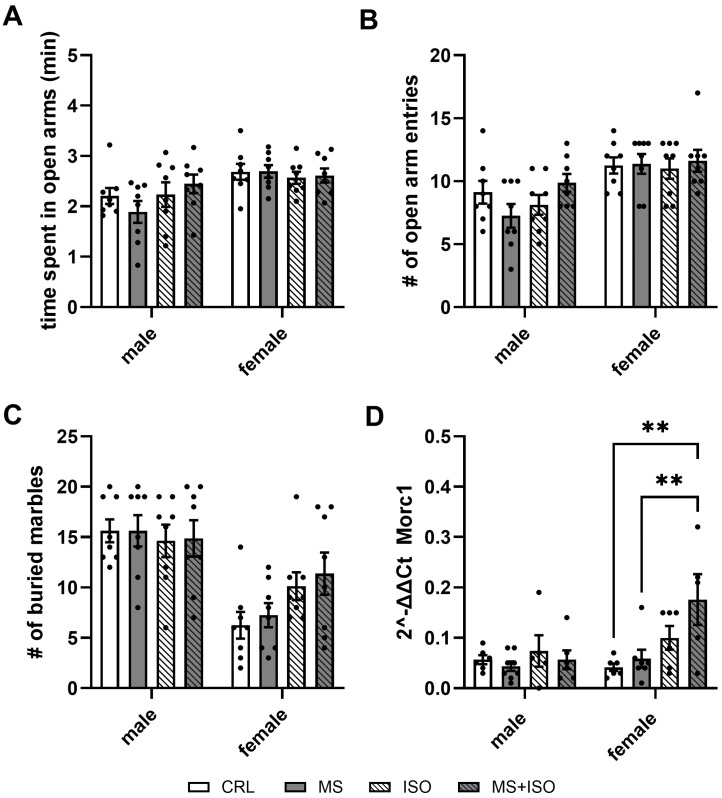
Behavioral and gene expression results for adults. Time spent in the open arms of the elevated plus-maze of a total of 5 min (**A**) and the total number of open-arm entries (**B**). The mean number of buried marbles after 15 min (**C**) and mean mPFC Morc1 2^−∆∆CT^ values are given per group (**D**). Mean values and SD are given per group. CRL = controls; MS = maternal separation; ISO = social isolation; MS + ISO = both stressors; ** *p* < 0.01.

Time spent in the open arm of the elevated plus-maze did differ between sexes (F (1, 56) = 13.2; *p* < 0.001), with females overall spending 17% more time in the open arms compared to males. No other main effects or interactions were found (ISO: F (1, 56) = 0.62; *p* = 0.44; MS: F (1, 56) = 0.01; *p* = 0.91; sex × ISO: F (1, 56) = 2.55; *p* = 0.12; sex × MS: F (1, 56) = 0.1, *p* = 0.75; ISO × MS: F (1, 56) = 1.34, *p* = 0.25; sex × ISO × MS: F (1, 56) = 1.02; *p* = 0.32) (Figure 4A). Similarly, there was a sex difference for the number of open-arm entries with females entering the open arms about 30% more often (F (1, 56) = 22.73; *p* < 0.001), and no other main effects or interactions present (ISO: F (1, 56) = 0.5; *p* = 0.48; MS: F (1, 56) = 0.08; *p* = 0.79; sex × ISO: F (1, 56) = 0.5; *p* = 0.48; sex × MS: F (1, 56) = 0.15, *p* = 0.7; ISO × MS: F (1, 56) = 3.3, *p* = 0.08; sex × ISO × MS: F (1, 56) = 1.9; *p* = 0.18) (Figure 4B).

The number of buried marbles was influenced by sex (F (1, 56) = 34.8; *p* < 0.001) and an interaction of sex × ISO (F (1, 56) = 4.98; *p* = 0.03). There were no main effects of MS (F (1, 56) = 0.33; *p* = 0.57 or ISO (F (1, 56) = 2.048; *p* = 0.16) nor any further interactions (sex × MS: F (1, 56) = 0.2; *p* = 0.65; ISO × MS: F (1, 56) = 0.01; *p* = 0.9; sex × ISO × MS: F (1, 56) < 0.0001; *p* > 0.99). Males buried nearly twice as many marbles as females. In males, isolation slightly reduced the number of buried marbles, while in females, isolation increased the number of buried marbles. However, the post-analysis revealed no significant effects between groups (Figure 4C).

There were significant main effects of sex (F (1, 41) = 5.42; *p* = 0.025), ISO (F (1, 41) = 10.91; *p* = 0.002) and an interaction of sex × ISO (F (1, 41) = 5.4; *p* = 0.025) for *Morc1* expression. No main effect for MS (F (1, 41) = 1; *p* = 0.32) or other interactions were found (sex × MS: F (1, 41) = 3.9; *p* = 0.055; ISO × MS: F (1, 41) = 0.77; *p* = 0.39; sex × ISO × MS: F (1, 41) = 1.04; *p* = 0.31). In females, the MS + ISO group showed higher 2^−∆∆CT^ values (lower *Morc1* expression) compared to the control (*p* = 0.001) and the MS (*p* = 0.003) group (Figure 4D).

## 4. Discussion

This study revealed different consequences of stressors during early life on anxiety-like behavior and *Morc1* mRNA levels in the mPFC. Interestingly, we report alternating effects depending on the developmental stage, highlighting the importance of timing for both exposure and phenotyping. In our study, juveniles showed significantly less anxiety-like behavior after MS in the elevated plus-maze than controls, with especially females demonstrating less anxiety-like behavior after MS. In adolescents, the main effect of sex was evident in the elevated plus-maze, with females spending more time in the open arm and showing about 20% more open-arm entries compared to males. ISO increased the time spent in the open arms in males but led to reduced entries in females. In the marble burying test, male adolescents exposed to MS, ISO, or both buried more marbles than controls. In females, only rats exposed to both stressors buried significantly more marbles than MS-exposed rats, indicating higher anxiety-like behavior. Animals tested in early adulthood demonstrated marked differences in anxiety-like behavior depending on sex, with males overall showing increased anxiety-like behavior compared to females. However, no effect of stress exposure on anxiety-like behavior was evident. MS has beneficial consequences in juvenility, but more profound effects arise after the second, later isolation stress in adolescence. The behavioral changes observed in juvenility and adolescence were not mirrored in neuronal *Morc1* mRNA levels. Even though no altered anxiety-like behavior was present in adults, alterations in *Morc1* expression were found in adult females with lower expression of *Morc1* after MS + social isolation compared to controls and MS. Possibly, group housing during adolescence attenuated the stress-induced alterations found in adolescence leading to no changes in behavior after early exposure in adulthood.

To our knowledge, this is the first study to assess anxiety-like behavior after MS in juveniles upon weaning. Thus, initially, it might seem striking that MS juveniles demonstrate less anxiety-like behavior than controls. However, daily isolation may render juveniles less anxious when exposed to new situations, such as in the test setting. Unexposed juveniles, in contrast, only experienced separation for weighting and, thus, might show more anxiety-like behavior in new situations. When maternal behavior is observed in MS dams, increased maternal care is found compared to control dams [26,60]. The increased maternal care upon reunion could protect against stress-induced consequences in juvenility, as high maternal care positively affects the pups’ stress response [61]. Here, early separation in the form of MS followed by a reunion phase might render females more explorative compared to unexposed controls.

The difference in behavioral alterations after exposure depending on sex found in adolescent rats may be driven by differences in brain development. Generally, sex differences in brain development have also been found in humans [62] and rodents [63]. A large population-based, longitudinal study observed that the volume increase in the frontal and parietal lobe gray matter peaks earlier in girls than boys during development [64]. Other studies found increased amygdala, thalamus, putamen, and insula volumes in boys compared to girls (for review, see [65]). Thus, it might be that consequences of early exposure become visible in altered anxiety-like behavior later in males than in females, given the sex-specific developmental trajectories.

Adult rats showed subtler behavioral implications after exposure (most differences did not reach significance). Potentially, group housing in adolescence attenuates stress-induced alterations. Therefore, group housing after exposure might attenuate behavioral differences found in adolescence. Interestingly, adult females exposed to social isolation seem to show less anxiety-like behavior, suggesting that the time spent in group housing did not attenuate the stressor-induced alterations. Generally, studies investigating the long-term effects of MS in adult rats show mixed results [66]. While some, including our study [54], revealed long-lasting changes in anxiety-like behavior in adult rats, others report no effect in adults measured with the elevated plus-maze test (for a review and meta-analysis, see [66]). A recent meta-analysis of 44 studies on the impact of MS on elevated plus-maze behavior in rats found that when adjusting for publication bias, overall, there was no significant association between MS and elevated plus-maze performance [66]. The authors furthermore showed that the age of testing had a significant effect with greater consequences of MS on exploratory behavior in the elevated plus-maze in younger animals [66].

The sex difference found in adults, with females showing generally less anxiety-like behavior than males, independent of exposure, is frequently reported [41,67,68]. Especially regarding MS, females may be more resilient to expressing phenotypic implications than males. Others also reported these sex differences and the potential resilience of females against MS [69]. Generally, differences in impairments of exposure to neurobiology and behavior have repeatedly been reported [29,33,40,41,68,70,71,72], underlining the importance of including both sexes. For example, Gildawie et al. (2021) found a sex-specific effect of early stress exposure in adult females with an opposing impact of MS and social isolation on hyperactivity and risk assessment behavior, as well as a reduction in PFC parvalbumin cells after cumulative stress [40].

Frequently, studies applying MS and ISO consecutively find different implications of cumulative compared to a single exposure [39,41], e.g., a weaker reduction in hippocampal plasticity and a more significant reduction in corticosterone levels after cumulative exposure compared to solely ISO [39]. In our study, significant differences were evident in the number of marbles buried after ISO or MS + ISO in adolescence compared to controls. Moreover, *Morc1* mRNA expression in adults decreased after exposure to MS + ISO or ISO, implying an essential effect of the timing of exposure rather than the overall length of exposure (as MS is usually conducted in the early days and ISO after weaning).

No long-lasting changes in anxiety-like behavior were evident in adults after MS or ISO. Group housing in late adolescence possibly attenuated the stress-induced alterations visible in adolescent rats. However, a recent meta-analysis of 44 studies on the effects of MS on elevated plus-maze behavior in rats revealed that studies report mixed results [66]. While some, including our study [54], revealed long-lasting changes in anxiety-like behavior in adult rats, others reported no effect in adults. When adjusting for publication bias, overall, there was no significant association between MS and elevated plus-maze performance [66]. The authors concluded that the age of testing had a significant effect with greater consequences of MS on exploratory behavior in the elevated plus-maze in younger animals [66].

At the same time, molecular changes in *Morc1* mRNA expression are evident in adults exposed to isolation, indicating long-lasting neuronal alterations, especially after stress exposure during early adolescence. No differences in *Morc1* mRNA expression were evident in juveniles and adolescents. These findings are in line with the maturation processes of the chosen target region (mPFC). The PFC has a comparably long maturation period and is, thus, highly vulnerable to stress during early adolescence, supposedly because of a protracted glucocorticoid response that continues into adulthood [4]. Therefore, it seems reasonable to presume that stress-induced changes in PFC development are most pronounced when exposure occurs in late childhood to early adolescence and are likely to persist into adulthood. In rats, it was demonstrated that PFC white matter is gained throughout adolescence [73], increasing the likelihood of long-lasting impairments. The sex differences in *Morc1* mRNA expression in adult females after MS + ISO suggest that females might be more prone to showing neurobiological alterations after cumulative exposure. For example, the combined volume measurement of the prelimbic and infralimbic cortices in rats shows a significant decrease between postnatal day 35 and postnatal day 90 only in females but not in male rats [74]. More precisely, female rats show a neuronal loss in the mPFC between postnatal days 35–45 with no further loss in adulthood, whereas, in male rats, a trend towards neuronal loss was shown throughout development (from postnatal day 20–90) [73]. These results could thus indicate that females, showing a greater neuronal loss in the mPFC during adolescence, are more vulnerable to exposure during that time, which leads to more profound neuronal alterations such as decreased *Morc1* expression in adulthood. Of note, in our previous study in human adults, we also report an effect of sex with women showing higher methylation of the CG site cg07090057 than men [19].

Long-lasting changes in gene expression that are induced by MS and demonstrate region-, age-, and sex-specific alterations are also reported for other genes; for example, the transcription factor orthodenticle homeobox 2 (*Otx2*), the glucocorticoid receptor gene *Nr3 c1,* the oxytocin receptor gene *Oxtr,* the serotonin receptor gene, and genes involved in the HPA axis.

In addition to *Morc1*, other genes have also been implicated in stress-related changes in gene expression, with MS inducing long-lasting, region-, age-, and sex-specific alterations across various genes. These changes have been observed in multiple genes, including the transcription factor *Otx2* (orthodenticle homeobox 2) [30], the glucocorticoid receptor gene (*Nr3 c1*) [75], the oxytocin receptor gene (*Oxtr*) [76], the serotonin receptor gene, and genes involved in the HPA axis [77,78,79].

## 5. Conclusions

Early-life stress exposure induced altered anxiety-like behavior with changes in directionality across development and sex. Interestingly, males seem to show more behavioral effects of stress exposure during adolescence, indicated by increased anxiety-like behavior, while females only show increased anxiety-like behavior after MS + ISO. After early-life stress, group housing attenuates the effects of early exposure. Furthermore, long-lasting implications of the late ISO stress on *Morc1* mRNA expression in the mPFC were evident in female adults. However, given the small sample size for the analyses, the results should be interpreted carefully and replicated in larger samples. The differences between the three developmental stages and sexes reinforce the need to include several ages and both sexes in all studies, as there seem to be multiple windows of vulnerability toward stress exposure.

## Figures and Tables

**Table 1:** Sample composition for the real-time PCR analysis. Number of animals included in the analyses per age when sacrificed and group. MS: maternal separation, CRL: controls, ISO: social isolation, P: postnatal day.

Age Tested	MS	CRL	ISO	MS + ISO
Juvenile (P22)	10	13	-	-
Adolescent (P42)	10	15	15	16
Adult (P62)	12	15	11	11

## Data Availability

Data will be made available upon request to the corresponding author.

## References

[1] Aisa B., Tordera R., Lasheras B., Del Río J., Ramírez M.J. (2008). Effects of Maternal Separation on Hypothalamic-Pituitary-Adrenal Responses, Cognition and Vulnerability to Stress in Adult Female Rats. Neuroscience.

[2] Frodl T., O’Keane V. (2013). How Does the Brain Deal with Cumulative Stress? A Review with Focus on Developmental Stress, HPA Axis Function and Hippocampal Structure in Humans. Neurobiol. Dis..

[3] Heim C., Newport D.J., Mletzko T., Miller A.H., Nemeroff C.B. (2008). The Link between Childhood Trauma and Depression: Insights from HPA Axis Studies in Humans. Psychoneuroendocrinology.

[4] Lupien S.J., McEwen B.S., Gunnar M.R., Heim C. (2009). Effects of Stress throughout the Lifespan on the Brain, Behaviour and Cognition. Nat. Rev. Neurosci..

[5] Hart H., Rubia K. (2012). Neuroimaging of Child Abuse: A Critical Review. Front. Hum. Neurosci..

[6] Carr C.P., Martins C.M.S., Stingel A.M., Lemgruber V.B., Juruena M.F. (2013). The Role of Early Life Stress in Adult Psychiatric Disorders: A Systematic Review According to Childhood Trauma Subtypes. J. Nerv. Ment. Dis..

[7] Teicher M.H., Samson J.A., Anderson C.M., Ohashi K. (2016). The Effects of Childhood Maltreatment on Brain Structure, Function and Connectivity. Nat. Rev. Neurosci..

[8] Kendler K.S., Kuhn J.W., Prescott C.A. (2004). Childhood Sexual Abuse, Stressful Life Events and Risk for Major Depression in Women. Psychol. Med..

[9] Mc Elroy S., Hevey D. (2014). Relationship between Adverse Early Experiences, Stressors, Psychosocial Resources and Wellbeing. Child Abuse Negl..

[10] Worlein J.M. (2014). Nonhuman Primate Models of Depression: Effects of Early Experience and Stress. ILAR J..

[11] Geronazzo-Alman L., Eisenberg R., Shen S., Duarte C.S., Musa G.J., Wicks J., Fan B., Doan T., Guffanti G., Bresnahan M. (2017). Cumulative Exposure to Work-Related Traumatic Events and Current Post-Traumatic Stress Disorder in New York City’s First Responders. Compr. Psychiatry.

[12] Suliman S., Mkabile S.G., Fincham D.S., Ahmed R., Stein D.J., Seedat S. (2009). Cumulative Effect of Multiple Trauma on Symptoms of Posttraumatic Stress Disorder, Anxiety, and Depression in Adolescents. Compr. Psychiatry.

[13] Belleau E.L., Treadway M.T., Pizzagalli D.A. (2019). The Impact of Stress and Major Depressive Disorder on Hippocampal and Medial Prefrontal Cortex Morphology. Biol. Psychiatry.

[14] Saxton K., Chyu L. (2020). Early Life Adversity Increases the Salience of Later Life Stress: An Investigation of Interactive Effects in the PSID. J. Dev. Orig. Health Dis..

[15] Aragam N., Wang K.-S., Pan Y. (2011). Genome-Wide Association Analysis of Gender Differences in Major Depressive Disorder in the Netherlands NESDA and NTR Population-Based Samples. J. Affect. Disord..

[16] Farrell C., O’Keane V. (2016). Epigenetics and the Glucocorticoid Receptor: A Review of the Implications in Depression. Psychiatry Res..

[17] Sullivan P.F., de Geus E.J.C., Willemsen G., James M.R., Smit J.H., Zandbelt T., Arolt V., Baune B.T., Blackwood D., Cichon S. (2009). Genome-Wide Association for Major Depressive Disorder: A Possible Role for the Presynaptic Protein Piccolo. Mol. Psychiatry.

[18] Mundorf A., Schmitz J., Hünten K., Fraenz C., Schlüter C., Genç E., Ocklenburg S., Freund N. (2021). MORC1 Methylation and BDI Are Associated with Microstructural Features of the Hippocampus and Medial Prefrontal Cortex. J. Affect. Disord..

[19] Mundorf A., Schmitz J., Güntürkün O., Freund N., Ocklenburg S. (2018). Methylation of MORC1: A Possible Biomarker for Depression?. J. Psychiatr. Res..

[20] Nieratschker V., Massart R., Gilles M., Luoni A., Suderman M.J., Krumm B., Meier S., Witt S.H., Nöthen M.M., Suomi S.J. (2014). MORC1 Exhibits Cross-Species Differential Methylation in Association with Early Life Stress as Well as Genome-Wide Association with MDD. Transl. Psychiatry.

[21] Thomas M., Coope A., Falkenberg C., Dunlop B.W., Czamara D., Provencal N., Craighead W.E., Mayberg H.S., Nemeroff C.B., Binder E.B. (2020). Investigation of MORC1 DNA Methylation as Biomarker of Early Life Stress and Depressive Symptoms. J. Psychiatr. Res..

[22] Razin A., Cedar H. (1991). DNA Methylation and Gene Expression. Microbiol. Rev..

[23] Moore L.D., Le T., Fan G. (2013). DNA Methylation and Its Basic Function. Neuropsychopharmacology.

[24] Mundorf A., Koch J., Kubitza N., Wagner S.C., Schmidt M., Gass P., Freund N. (2021). Morc1 as a Potential New Target Gene in Mood Regulation: When and Where to Find in the Brain. Exp. Brain Res..

[25] Schmidt M., Brandwein C., Luoni A., Sandrini P., Calzoni T., Deuschle M., Cirulli F., Riva M.A., Gass P. (2016). Morc1 Knockout Evokes a Depression-like Phenotype in Mice. Behav. Brain Res..

[26] Bölükbas I., Mundorf A., Freund N. (2020). Maternal Separation in Rats Induces Neurobiological and Behavioral Changes on the Maternal Side. Sci. Rep..

[27] Lehmann J., Feldon J. (2000). Long-Term Biobehavioral Effects of Maternal Separation in the Rat: Consistent or Confusing?. Rev. Neurosci..

[28] Tractenberg S.G., Levandowski M.L., de Azeredo L.A., Orso R., Roithmann L.G., Hoffmann E.S., Brenhouse H., Grassi-Oliveira R. (2016). An Overview of Maternal Separation Effects on Behavioural Outcomes in Mice: Evidence from a Four-Stage Methodological Systematic Review. Neurosci. Biobehav. Rev..

[29] Freund N., Thompson B.S., Denormandie J., Vaccarro K., Andersen S.L. (2013). Windows of Vulnerability: Maternal Separation, Age, and Fluoxetine on Adolescent Depressive-like Behavior in Rats. Neuroscience.

[30] Peña C.J., Kronman H.G., Walker D.M., Cates H.M., Bagot R.C., Purushothaman I., Issler O., Loh Y.-H.E., Leong T., Kiraly D.D. (2017). Early Life Stress Confers Lifelong Stress Susceptibility in Mice via Ventral Tegmental Area OTX2. Science.

[31] Zhou L., Wu Z., Wang G., Xiao L., Wang H., Sun L., Xie Y. (2020). Long-Term Maternal Separation Potentiates Depressive-like Behaviours and Neuroinflammation in Adult Male C57/BL6J Mice. Pharmacol. Biochem. Behav..

[32] Cui Y., Cao K., Lin H., Cui S., Shen C., Wen W., Mo H., Dong Z., Bai S., Yang L. (2020). Early-Life Stress Induces Depression-Like Behavior and Synaptic-Plasticity Changes in a Maternal Separation Rat Model: Gender Difference and Metabolomics Study. Front. Pharmacol..

[33] Honeycutt J.A., Demaestri C., Peterzell S., Silveri M.M., Cai X., Kulkarni P., Cunningham M.G., Ferris C.F., Brenhouse H.C. (2020). Altered Corticolimbic Connectivity Reveals Sex-Specific Adolescent Outcomes in a Rat Model of Early Life Adversity. eLife.

[34] Brenhouse H.C., Lukkes J.L., Andersen S.L. (2013). Early Life Adversity Alters the Developmental Profiles of Addiction-Related Prefrontal Cortex Circuitry. Brain Sci..

[35] Andersen S.L., Teicher M.H. (2004). Delayed Effects of Early Stress on Hippocampal Development. Neuropsychopharmacol. Off. Publ. Am. Coll. Neuropsychopharmacol..

[36] Gildawie K.R., Honeycutt J.A., Brenhouse H.C. (2020). Region-Specific Effects of Maternal Separation on Perineuronal Net and Parvalbumin-Expressing Interneuron Formation in Male and Female Rats. Neuroscience.

[37] Mumtaz F., Khan M.I., Zubair M., Dehpour A.R. (2018). Neurobiology and Consequences of Social Isolation Stress in Animal Model—A Comprehensive Review. Biomed. Pharmacother..

[38] Weiss I.C., Pryce C.R., Jongen-Rêlo A.L., Nanz-Bahr N.I., Feldon J. (2004). Effect of Social Isolation on Stress-Related Behavioural and Neuroendocrine State in the Rat. Behav. Brain Res..

[39] Biggio F., Pisu M.G., Garau A., Boero G., Locci V., Mostallino M.C., Olla P., Utzeri C., Serra M. (2014). Maternal Separation Attenuates the Effect of Adolescent Social Isolation on HPA Axis Responsiveness in Adult Rats. Eur. Neuropsychopharmacol. J. Eur. Coll. Neuropsychopharmacol..

[40] Gildawie K.R., Ryll L.M., Hexter J.C., Peterzell S., Valentine A.A., Brenhouse H.C. (2021). A Two-Hit Adversity Model in Developing Rats Reveals Sex-Specific Impacts on Prefrontal Cortex Structure and Behavior. Dev. Cogn. Neurosci..

[41] Jaric I., Rocks D., Cham H., Herchek A., Kundakovic M. (2019). Sex and Estrous Cycle Effects on Anxiety- and Depression-Related Phenotypes in a Two-Hit Developmental Stress Model. Front. Mol. Neurosci..

[42] Lukkes J.L., Mokin M.V., Scholl J.L., Forster G.L. (2009). Adult Rats Exposed to Early-Life Social Isolation Exhibit Increased Anxiety and Conditioned Fear Behavior, and Altered Hormonal Stress Responses. Horm. Behav..

[43] Vargas J., Junco M., Gomez C., Lajud N. (2016). Early Life Stress Increases Metabolic Risk, HPA Axis Reactivity, and Depressive-Like Behavior When Combined with Postweaning Social Isolation in Rats. PLoS ONE.

[44] Solmi M., Radua J., Olivola M., Croce E., Soardo L., Salazar de Pablo G., Il Shin J., Kirkbride J.B., Jones P., Kim J.H. (2021). Age at Onset of Mental Disorders Worldwide: Large-Scale Meta-Analysis of 192 Epidemiological Studies. Mol. Psychiatry.

[45] De Brouwer G., Fick A., Harvey B.H., Wolmarans D.W. (2019). A Critical Inquiry into Marble-Burying as a Preclinical Screening Paradigm of Relevance for Anxiety and Obsessive-Compulsive Disorder: Mapping the Way Forward. Cogn. Affect. Behav. Neurosci..

[46] Pellow S., Chopin P., File S.E., Briley M. (1985). Validation of Open: Closed Arm Entries in an Elevated plus-Maze as a Measure of Anxiety in the Rat. J. Neurosci. Methods.

[47] Poling A., Cleary J., Monaghan M. (1981). Burying by Rats in Response to Aversive and Nonaversive Stimuli. J. Exp. Anal. Behav..

[48] Jarrar Q., Ayoub R., Alhussine K., Goh K.W., Moshawih S., Ardianto C., Goh B.H., Ming L.C. (2022). Prolonged Maternal Separation Reduces Anxiety State and Increases Compulsive Burying Activity in the Offspring of BALB/c Mice. J. Pers. Med..

[49] Pellow S., File S.E. (1986). Anxiolytic and Anxiogenic Drug Effects on Exploratory Activity in an Elevated Plus-Maze: A Novel Test of Anxiety in the Rat. Pharmacol. Biochem. Behav..

[50] Andersen S.L., Teicher M.H. (2008). Stress, Sensitive Periods and Maturational Events in Adolescent Depression. Trends Neurosci..

[51] Chocyk A., Majcher-Maślanka I., Dudys D., Przyborowska A., Wędzony K. (2013). Impact of Early-Life Stress on the Medial Prefrontal Cortex Functions—A Search for the Pathomechanisms of Anxiety and Mood Disorders. Pharmacol. Rep. PR.

[52] Faul F., Erdfelder E., Lang A.-G., Buchner A. (2007). G*Power 3: A Flexible Statistical Power Analysis Program for the Social, Behavioral, and Biomedical Sciences. Behav. Res. Methods.

[53] Paxinos G., Watson C. (2006). The Rat Brain in Stereotaxic Coordinates.

[54] Abraham M., Schmerder K., Hedtstück M., Bösing K., Mundorf A., Freund N. (2023). Maternal Separation and Its Developmental Consequences on Anxiety and Parvalbumin Interneurons in the Amygdala. J. Neural Transm..

[55] Mundorf A., Matsui H., Ocklenburg S., Freund N. (2020). Asymmetry of Turning Behavior in Rats Is Modulated by Early Life Stress. Behav. Brain Res..

[56] Mundorf A., Kubitza N., Hünten K., Matsui H., Juckel G., Ocklenburg S., Freund N. (2021). Maternal Immune Activation Leads to Atypical Turning Asymmetry and Reduced DRD2 mRNA Expression in a Rat Model of Schizophrenia. Behav. Brain Res..

[57] De Boer M.E., de Boer T.E., Mariën J., Timmermans M.J., Nota B., van Straalen N.M., Ellers J., Roelofs D. (2009). Reference Genes for QRT-PCR Tested under Various Stress Conditions in Folsomia Candida and Orchesella Cincta (Insecta, Collembola). BMC Mol. Biol..

[58] Zainuddin A., Chua K.H., Abdul Rahim N., Makpol S. (2010). Effect of Experimental Treatment on GAPDH mRNA Expression as a Housekeeping Gene in Human Diploid Fibroblasts. BMC Mol. Biol..

[59] Derveaux S., Vandesompele J., Hellemans J. (2010). How to Do Successful Gene Expression Analysis Using Real-Time PCR. Methods San Diego Calif.

[60] Alves R.L., Portugal C.C., Summavielle T., Barbosa F., Magalhães A. (2020). Maternal Separation Effects on Mother Rodents’ Behaviour: A Systematic Review. Neurosci. Biobehav. Rev..

[61] Weaver I.C.G., Cervoni N., Champagne F.A., D’Alessio A.C., Sharma S., Seckl J.R., Dymov S., Szyf M., Meaney M.J. (2004). Epigenetic Programming by Maternal Behavior. Nat. Neurosci..

[62] Kaczkurkin A.N., Raznahan A., Satterthwaite T.D. (2019). Sex Differences in the Developing Brain: Insights from Multimodal Neuroimaging. Neuropsychopharmacology.

[63] Premachandran H., Zhao M., Arruda-Carvalho M. (2020). Sex Differences in the Development of the Rodent Corticolimbic System. Front. Neurosci..

[64] Lenroot R.K., Gogtay N., Greenstein D.K., Wells E.M., Wallace G.L., Clasen L.S., Blumenthal J.D., Lerch J., Zijdenbos A.P., Evans A.C. (2007). Sexual Dimorphism of Brain Developmental Trajectories during Childhood and Adolescence. NeuroImage.

[65] Peper J.S., Brouwer R.M., Schnack H.G., van Baal G.C., van Leeuwen M., van den Berg S.M., Delemarre-Van de Waal H.A., Boomsma D.I., Kahn R.S., Hulshoff Pol H.E. (2009). Sex Steroids and Brain Structure in Pubertal Boys and Girls. Psychoneuroendocrinology.

[66] Wang D., Levine J.L.S., Avila-Quintero V., Bloch M., Kaffman A. (2020). Systematic Review and Meta-Analysis: Effects of Maternal Separation on Anxiety-like Behavior in Rodents. Transl. Psychiatry.

[67] Kokras N., Dalla C. (2014). Sex Differences in Animal Models of Psychiatric Disorders. Br. J. Pharmacol..

[68] Leussis M.P., Freund N., Brenhouse H.C., Thompson B.S., Andersen S.L. (2012). Depressive-like Behavior in Adolescents after Maternal Separation: Sex Differences, Controllability, and GABA. Dev. Neurosci..

[69] Dimatelis J.J., Vermeulen I.M., Bugarith K., Stein D.J., Russell V.A. (2016). Female Rats Are Resistant to Developing the Depressive Phenotype Induced by Maternal Separation Stress. Metab. Brain Dis..

[70] Ellis S.N., Honeycutt J.A. (2021). Sex Differences in Affective Dysfunction and Alterations in Parvalbumin in Rodent Models of Early Life Adversity. Front. Behav. Neurosci..

[71] Mundorf A., Knorr A., Mezö C., Klein C., Beyer D.K., Fallgatter A.J., Schwarz M., Freund N. (2019). Lithium and Glutamine Synthetase: Protective Effects Following Stress. Psychiatry Res..

[72] Schroeder A., Notaras M., Du X., Hill R.A. (2018). On the Developmental Timing of Stress: Delineating Sex-Specific Effects of Stress across Development on Adult Behavior. Brain Sci..

[73] Willing J., Juraska J.M. (2015). The Timing of Neuronal Loss across Adolescence in the Medial Prefrontal Cortex of Male and Female Rats. Neuroscience.

[74] Markham J.A., Morris J.R., Juraska J.M. (2007). Neuron Number Decreases in the Rat Ventral, but Not Dorsal, Medial Prefrontal Cortex between Adolescence and Adulthood. Neuroscience.

[75] Tran C.H., Shannon Weickert C., Weickert T.W., Sinclair D. (2022). Early Life Stress Alters Expression of Glucocorticoid Stress Response Genes and Trophic Factor Transcripts in the Rodent Basal Ganglia. Int. J. Mol. Sci..

[76] Lukas M., Bredewold R., Neumann I.D., Veenema A.H. (2010). Maternal Separation Interferes with Developmental Changes in Brain Vasopressin and Oxytocin Receptor Binding in Male Rats. Neuropharmacology.

[77] Comasco E., Schijven D., de Maeyer H., Vrettou M., Nylander I., Sundström-Poromaa I., Olivier J.D.A. (2019). Constitutive Serotonin Transporter Reduction Resembles Maternal Separation with Regard to Stress-Related Gene Expression. ACS Chem. Neurosci..

[78] Own L.S., Iqbal R., Patel P.D. (2013). Maternal Separation Alters Serotonergic and HPA Axis Gene Expression Independent of Separation Duration in Mice. Brain Res..

[79] Nishi M. (2020). Effects of Early-Life Stress on the Brain and Behaviors: Implications of Early Maternal Separation in Rodents. Int. J. Mol. Sci..

